# Olanzapinium dipicrate

**DOI:** 10.1107/S1600536813000640

**Published:** 2013-01-12

**Authors:** C. N. Kavitha, Jerry P. Jasinski, Amanda C. Keeley, H. S. Yathirajan, A. S. Dayananda

**Affiliations:** a Department of Studies in Chemistry, University of Mysore, Manasagangotri, Mysore 570 006, India; b Department of Chemistry, Keene State College, 229 Main Street, Keene, NH 03435-2001, USA

## Abstract

The asymmetric unit of the title salt [systematic name: 2-methyl-4-(4-methyl­piperazin-4-ium-1-yl)-10*H*-thieno[2,3-*b*][1,5]benzodiazepinium bis­(2,4,6-trinitro­phenolate)], C_17_H_22_N_4_S^2+^·2C_6_H_2_N_3_O_7_^−^, consists of a diprotonated olanzapinium cation and two independent picrate anions. In the cation, the piperazine ring adopts a distorted chair conformation and contains a positively charged N atom with quaternary character and the N atom in the seven-membered 1,5-diazepine ring, which adopts a boat configuration, is also protonated. The dihedral angle between the benzene and thiene rings flanking the diazepine ring is 58.8 (1)°. In one of the picrate anions, a nitro group is disordered over two sets of sites in a 0.748 (5):0.252 (5) ratio, and the benzene ring has a flat envelope conformation with the O^−^ C atom displaced from the mean plane of the other five C atoms [maximum deviation 0.0151 (14) Å] by 0.1449 (14) Å. In the crystal, N—H⋯O hydrogen bonds and weak inter­molecular C—H⋯S and C—H⋯O inter­actions link the components, forming a three-dimensional network.

## Related literature

For the use of olanzapine in the management of bipolar disorder, see: Narasimhan *et al.* (2007 ▶) and for toxicity and fatality associated with its overdose, see: Chue & Singer (2003 ▶). For related structures, see: Capuano *et al.* (2003 ▶); Wawrzycka-Gorczyca *et al.* (2004*a* ▶,*b* ▶, 2006 ▶); Ravikumar *et al.* (2005 ▶); Thakuria & Nangia (2011*a* ▶,*b* ▶). For puckering parameters, see: Cremer & Pople (1975 ▶). For standard bond lengths, see: Allen *et al.* (1987 ▶).
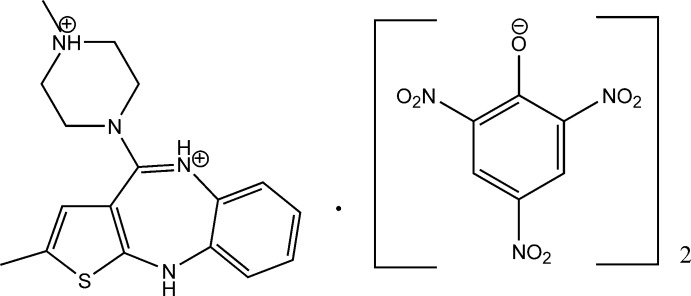


## Experimental

### 

#### Crystal data

C_17_H_22_N_4_S^2+^·2C_6_H_2_N_3_O_7_^−^
*M* *_r_* = 770.66
Orthorhombic, 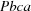

*a* = 22.1660 (4) Å
*b* = 12.7349 (2) Å
*c* = 23.3951 (4) Å
*V* = 6604.04 (19) Å^3^
*Z* = 8
Cu *K*α radiation
μ = 1.65 mm^−1^
*T* = 173 K
0.28 × 0.22 × 0.12 mm

#### Data collection

Agilent Xcalibur (Eos, Gemini) diffractometer
Absorption correction: multi-scan (*CrysAlis PRO* and *CrysAlis RED*; Agilent, 2012 ▶) *T*_min_ = 0.702, *T*_max_ = 1.000
44193 measured reflections
6524 independent reflections
5508 reflections with *I* > 2σ(*I*)
*R*_int_ = 0.041

#### Refinement

*R*[*F* ^2^ > 2σ(*F* ^2^)] = 0.038
*wR*(*F* ^2^) = 0.107
*S* = 1.03
6524 reflections
506 parameters
H atoms treated by a mixture of independent and constrained refinement
Δρ_max_ = 0.29 e Å^−3^
Δρ_min_ = −0.25 e Å^−3^

### 

Data collection: *CrysAlis PRO* (Agilent, 2012 ▶); cell refinement: *CrysAlis PRO*; data reduction: *CrysAlis RED* (Agilent, 2012 ▶); program(s) used to solve structure: *SHELXS97* (Sheldrick, 2008 ▶); program(s) used to refine structure: *SHELXL97* (Sheldrick, 2008 ▶); molecular graphics: *SHELXTL* (Sheldrick, 2008 ▶); software used to prepare material for publication: *SHELXTL*.

## Supplementary Material

Crystal structure: contains datablock(s) global, I. DOI: 10.1107/S1600536813000640/bv2217sup1.cif

Structure factors: contains datablock(s) I. DOI: 10.1107/S1600536813000640/bv2217Isup2.hkl

Supplementary material file. DOI: 10.1107/S1600536813000640/bv2217Isup3.cml

Additional supplementary materials:  crystallographic information; 3D view; checkCIF report

## Figures and Tables

**Table 1:** Hydrogen-bond geometry (Å, °)

*D*—H⋯*A*	*D*—H	H⋯*A*	*D*⋯*A*	*D*—H⋯*A*
N2—H2⋯O1*A*	0.872 (17)	1.848 (17)	2.7086 (17)	168.5 (16)
N4—H4⋯O1*B*	0.926 (19)	1.859 (19)	2.6612 (17)	143.6 (17)
N4—H4⋯O7*B*	0.926 (19)	2.449 (19)	3.1714 (19)	135.0 (15)
N1—H1⋯O5*B* ^i^	0.852 (19)	2.40 (2)	3.1395 (18)	145.9 (17)
C5*B*—H5*B*⋯S1^ii^	0.93	2.87	3.6225 (15)	139
C8—H8⋯O3*A* ^iii^	0.93	2.48	3.349 (2)	156
C12—H12*A*⋯O5*A* ^iv^	0.97	2.23	3.0970 (19)	149
C17—H17*B*⋯O6*A* ^iv^	0.96	2.43	3.207 (2)	138

Symmetry codes: (i) 
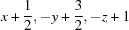
; (ii) 
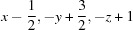
; (iii) 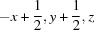; (iv) 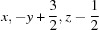.

**Fig. 1.**
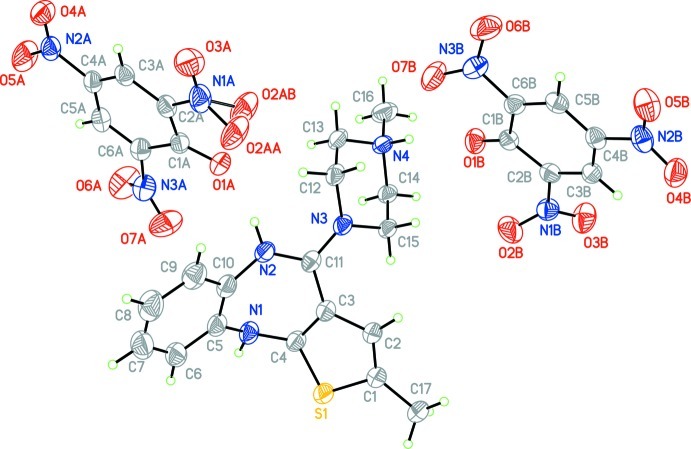
Molecular structure of the title compound showing the atom labeling scheme of the olanzapinium and picrate moieties in the asymmetric unit and 50% probability displacement ellipsoids.

**Fig. 2.**
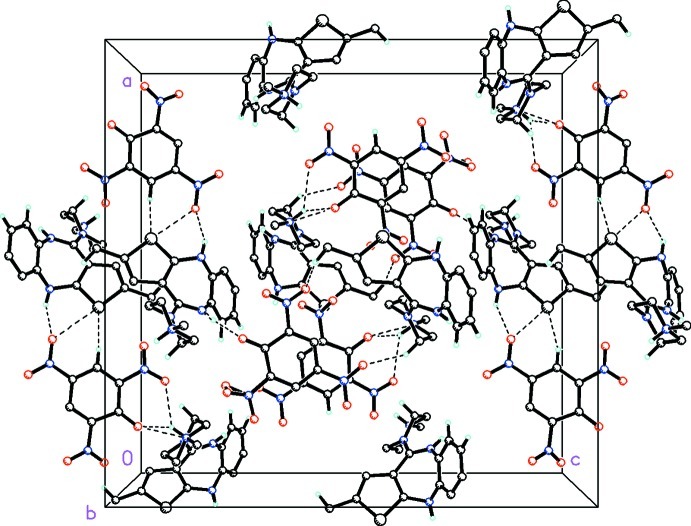
Packing diagram of the title compound viewed along the b axis. Dashed lines indicate weak N—H···O intramolecular hydrogen bonds and weak N—H···O, C—H···S, C—H···O intermolecular interactions forming infinite 1-D chains along [010]. The remaining H atoms have been removed for clarity.

## supplementary crystallographic information

### Comment

Olanzapine is an atypical antipsychotic currently with indications for the treatment of schizophrenia, acute mania and the prevention of relapse in bipolar disorder. Olanzapine is structurally similar to clozapine, but is classified as a thienobenzodiazepine. Reviews on olanzapine in the management of bipolar disorders (Narasimhan *et al.*, 2007) and olanzapine associated toxicity and fatality in overdose (Chue & Singer, 2003) have been published. The crystal structures of 2-methyl-4-(4-methylpiperazin-1-yl)-10H-thieno[2,3-b][1,5] benzodiazepine methanol solvate monohydrate (Capuano *et al.*, 2003), polymorphic form II of 2-methyl-4-(4-methyl-1-piperazinyl)-10H-thieno[2,3-b] [1,5]benzodiazepine, (Wawrzycka-Gorczyca *et al.*, 2004*a*), 2-methyl-4-(4-methyl-1-piperazinyl)-10H-thieno[2,3-b][1,5] benzodiazepine methanol solvate (Wawrzycka-Gorczyca *et al.*, 2004*b*), olazipinium nicotinate (Ravikumar *et al.*, 2005), olanzapine and its solvates (Wawrzycka-Gorczyca *et al.*, 2006), highly soluble olanzapinium maleate crystalline salts (Thakuria & Nangia, 2011*a*) and polymorphic form IV of olanzapine (Thakuria & Nangia, 2011*b*) have been reported. In view of the importance of olanzapine, this paper reports the crystal structure of the title salt, (I), C_17_H_22_N_4_S^+2^. 2 C_6_H_2_N_3_O_7_^-^.

The asymmetric unit in (I) consists of a diprotonated olanzapinium cation where one N atom in the piperazine ring and another N atom in the seven-membered 1,5-diazepine ring in a boat configuration are protonated and two independent picrate anions (A & B)(Fig. 1). The six-membered piperazine ring, N3/C12/C13/N4/C14/C15, adopts a distorted chair conformation with puckering parameters Q = 0.5641 (15)Å, θ = 177.35 (16)°, φ = 69 (3)° (Cremer & Pople (1975) and contains one positively charged N atom with quaternary character. The dihedral angle between the benzene and thiene rings flanking the diazepine ring is 58.7 (9)°. In picrate anion A, the benzene ring adopts a distorted screw-boat configuration with puckering parameters Q = 0.1022 (16)Å, θ = 66.9 (9)°, φ = 354.8 (10)°. Nitro atom O2AA is disordered [occupancy 0.748 (5):0.252 (5)]. The mean plane of the N3A-O6A-O7A group is twisted by 26.2 (3)° with that of the benzene ring. In picrate anion B the mean planes of the N1B-O2B-O3B and N3B-O6B-O7B nitro groups are twisted by 41.3 (1)° and 20.2 (4)° with that of the benzene ring. Bond lengths are in normal ranges (Allen *et al.*, (1987). In the crystal, N—H···O intramolecular hydrogen bonds and weak N—H···O, C—H···S, C—H···O intermolecular interactions (Table 1) and π–π stacking interactions (Centroid Cg4—Centroid Cg5; 3.7378 (8)Å; Cg4 = C1A–C6A and Cg5 = C1B–C6B] are observed forming infinite 1-D chains along [010] (Fig. 2).

### Experimental

Olanzapine (3.128 g, 0.01 mol) and picric acid (2.29 g, 0.01 mol) were dissolved in 10 ml of toluene and stirred over a heating magnetic stirrer for few minutes (330 K). The mixture was kept aside for two days at room temperature. The salt formed was filtered & dried. The compound was recrystallized from (1:1) toluene & DMF by slow evaporation (m.p: 323–325 K).

### Refinement

H1, H2 and H4 were located by a difference map and refined isotropically. All the remaining H atoms were placed in their calculated positions and then refined using the riding model with Atom—H lengths of 0.93Å (CH), 0.97Å (CH_2_) or 0.96å (CH_3_). Isotropic displacement parameters for these atoms were set to 1.19-1.21 (CH, CH_2_) or 1.50 (CH_3_) times *U*_eq_ of the parent atom.

### Crystal data

**Table:**

C_17_H_22_N_4_S^2+^·2C_6_H_2_N_3_O_7_^−^	*F*(000) = 3184
*M**_r_* = 770.66	*D*_x_ = 1.550 Mg m^−^^3^
Orthorhombic, *P**b**c**a*	Cu *K*α radiation, λ = 1.54184 Å
Hall symbol: -P 2ac 2ab	Cell parameters from 14060 reflections
*a* = 22.1660 (4) Å	θ = 3.5–72.4°
*b* = 12.7349 (2) Å	µ = 1.65 mm^−^^1^
*c* = 23.3951 (4) Å	*T* = 173 K
*V* = 6604.04 (19) Å^3^	Chunk, orange
*Z* = 8	0.28 × 0.22 × 0.12 mm

### Data collection

**Table:**

Agilent Xcalibur (Eos, Gemini) diffractometer	6524 independent reflections
Radiation source: Enhance (Cu) X-ray Source	5508 reflections with *I* > 2σ(*I*)
Graphite monochromator	*R*_int_ = 0.041
Detector resolution: 16.0416 pixels mm^-1^	θ_max_ = 72.5°, θ_min_ = 3.8°
ω scans	*h* = −27→18
Absorption correction: multi-scan (*CrysAlis PRO* and *CrysAlis RED*; Agilent, 2012)	*k* = −15→15
*T*_min_ = 0.702, *T*_max_ = 1.000	*l* = −28→28
44193 measured reflections	

### Refinement

**Table:**

Refinement on *F*^2^	Secondary atom site location: difference Fourier map
Least-squares matrix: full	Hydrogen site location: inferred from neighbouring sites
*R*[*F*^2^ > 2σ(*F*^2^)] = 0.038	H atoms treated by a mixture of independent and constrained refinement
*wR*(*F*^2^) = 0.107	*w* = 1/[σ^2^(*F*_o_^2^) + (0.0538*P*)^2^ + 2.5536*P*] where *P* = (*F*_o_^2^ + 2*F*_c_^2^)/3
*S* = 1.03	(Δ/σ)_max_ = 0.001
6524 reflections	Δρ_max_ = 0.29 e Å^−^^3^
506 parameters	Δρ_min_ = −0.25 e Å^−^^3^
0 restraints	Extinction correction: *SHELXL97* (Sheldrick, 2008), Fc^*^=kFc[1+0.001xFc^2^λ^3^/sin(2θ)]^-1/4^
Primary atom site location: structure-invariant direct methods	Extinction coefficient: 0.00018 (3)

### Special details

**Table:**

Geometry. All esds (except the esd in the dihedral angle between two l.s. planes) are estimated using the full covariance matrix. The cell esds are taken into account individually in the estimation of esds in distances, angles and torsion angles; correlations between esds in cell parameters are only used when they are defined by crystal symmetry. An approximate (isotropic) treatment of cell esds is used for estimating esds involving l.s. planes.
Refinement. Refinement of *F*^2^ against ALL reflections. The weighted *R*-factor *wR* and goodness of fit *S* are based on *F*^2^, conventional *R*-factors *R* are based on *F*, with *F* set to zero for negative *F*^2^. The threshold expression of *F*^2^ > σ(*F*^2^) is used only for calculating *R*-factors(gt) *etc*. and is not relevant to the choice of reflections for refinement. *R*-factors based on *F*^2^ are statistically about twice as large as those based on *F*, and *R*- factors based on ALL data will be even larger.

### Fractional atomic coordinates and isotropic or equivalent isotropic displacement parameters (Å^2^)

**Table:**

	*x*	*y*	*z*	*U*_iso_*/*U*_eq_	Occ. (<1)
S1	0.579266 (18)	0.92049 (4)	0.563737 (17)	0.04414 (11)	
N1	0.53681 (6)	0.91960 (11)	0.67285 (5)	0.0387 (3)	
H1	0.5726 (9)	0.9405 (16)	0.6797 (8)	0.051 (5)*	
N2	0.41626 (6)	0.85066 (10)	0.67800 (5)	0.0348 (3)	
H2	0.3945 (7)	0.8196 (13)	0.7041 (7)	0.031 (4)*	
N3	0.40406 (5)	0.70712 (10)	0.61938 (5)	0.0331 (3)	
N4	0.38127 (6)	0.48631 (11)	0.63699 (5)	0.0367 (3)	
H4	0.3563 (8)	0.4761 (15)	0.6057 (8)	0.051 (5)*	
C1	0.53417 (8)	0.86715 (13)	0.51046 (6)	0.0405 (4)	
C2	0.48250 (7)	0.82924 (12)	0.53276 (6)	0.0360 (3)	
H2A	0.4522	0.7992	0.5106	0.043*	
C3	0.47835 (6)	0.83939 (12)	0.59394 (6)	0.0327 (3)	
C4	0.52808 (7)	0.88977 (12)	0.61615 (6)	0.0347 (3)	
C5	0.49176 (7)	0.98865 (13)	0.69407 (6)	0.0375 (3)	
C6	0.50589 (9)	1.08776 (14)	0.71524 (7)	0.0472 (4)	
H6	0.5456	1.1114	0.7148	0.057*	
C7	0.46052 (11)	1.15108 (15)	0.73700 (8)	0.0585 (5)	
H7	0.4701	1.2161	0.7526	0.070*	
C8	0.40162 (11)	1.11827 (15)	0.73559 (9)	0.0603 (5)	
H8	0.3714	1.1618	0.7496	0.072*	
C9	0.38688 (9)	1.02055 (14)	0.71346 (7)	0.0487 (4)	
H9	0.3468	0.9988	0.7123	0.058*	
C10	0.43210 (7)	0.95562 (12)	0.69310 (6)	0.0372 (3)	
C11	0.43149 (6)	0.79801 (12)	0.63109 (6)	0.0310 (3)	
C12	0.34715 (6)	0.67087 (13)	0.64582 (6)	0.0368 (3)	
H12A	0.3165	0.6636	0.6164	0.044*	
H12B	0.3333	0.7231	0.6730	0.044*	
C13	0.35504 (7)	0.56706 (14)	0.67612 (6)	0.0410 (4)	
H13A	0.3814	0.5764	0.7088	0.049*	
H13B	0.3162	0.5429	0.6900	0.049*	
C14	0.43908 (6)	0.52558 (13)	0.61187 (6)	0.0346 (3)	
H14A	0.4551	0.4736	0.5856	0.042*	
H14B	0.4684	0.5360	0.6421	0.042*	
C15	0.42908 (6)	0.62748 (12)	0.58063 (6)	0.0320 (3)	
H15A	0.4671	0.6523	0.5651	0.038*	
H15B	0.4015	0.6162	0.5491	0.038*	
C16	0.39062 (9)	0.38329 (15)	0.66601 (8)	0.0541 (5)	
H16A	0.4068	0.3337	0.6392	0.081*	
H16B	0.3528	0.3579	0.6804	0.081*	
H16C	0.4184	0.3920	0.6972	0.081*	
C17	0.55665 (9)	0.86961 (16)	0.45003 (7)	0.0536 (5)	
H17A	0.5706	0.9390	0.4410	0.080*	
H17B	0.5245	0.8508	0.4245	0.080*	
H17C	0.5892	0.8206	0.4458	0.080*	
O1A	0.35350 (6)	0.77573 (10)	0.76857 (5)	0.0494 (3)	
O2AA	0.24618 (11)	0.8297 (2)	0.72987 (8)	0.0643 (7)	0.748 (5)
O2AB	0.2333 (4)	0.7808 (8)	0.7354 (3)	0.0643 (7)	0.252 (5)
O3A	0.17093 (6)	0.82511 (12)	0.79120 (5)	0.0590 (4)	
O4A	0.20275 (6)	0.83164 (10)	0.98965 (5)	0.0481 (3)	
O5A	0.29258 (7)	0.83455 (13)	1.02399 (5)	0.0632 (4)	
O6A	0.46450 (6)	0.80267 (14)	0.90134 (6)	0.0729 (4)	
O7A	0.45449 (6)	0.86082 (14)	0.81632 (6)	0.0703 (4)	
N1A	0.22493 (7)	0.81934 (12)	0.77957 (6)	0.0451 (3)	
N2A	0.25736 (7)	0.83171 (10)	0.98370 (5)	0.0385 (3)	
N3A	0.43342 (7)	0.82737 (12)	0.86039 (6)	0.0463 (3)	
C1A	0.33162 (7)	0.80385 (11)	0.81504 (6)	0.0349 (3)	
C2A	0.26786 (7)	0.81740 (11)	0.82648 (6)	0.0335 (3)	
C3A	0.24387 (7)	0.82569 (11)	0.88050 (6)	0.0322 (3)	
H3A	0.2023	0.8300	0.8857	0.039*	
C4A	0.28246 (7)	0.82754 (11)	0.92697 (6)	0.0326 (3)	
C5A	0.34468 (7)	0.82687 (11)	0.91966 (6)	0.0355 (3)	
H5A	0.3702	0.8311	0.9512	0.043*	
C6A	0.36793 (7)	0.82002 (12)	0.86602 (6)	0.0359 (3)	
O1B	0.35005 (5)	0.40692 (10)	0.53601 (5)	0.0439 (3)	
O2B	0.43648 (6)	0.46565 (12)	0.45828 (6)	0.0636 (4)	
O3B	0.42526 (6)	0.35698 (12)	0.38873 (6)	0.0590 (4)	
O4B	0.23268 (6)	0.43743 (10)	0.29528 (5)	0.0495 (3)	
O5B	0.15172 (5)	0.44113 (10)	0.34730 (5)	0.0509 (3)	
O6B	0.16618 (6)	0.40258 (13)	0.54990 (6)	0.0642 (4)	
O7B	0.24767 (6)	0.45166 (13)	0.59228 (5)	0.0607 (4)	
N1B	0.40510 (6)	0.41103 (11)	0.42742 (6)	0.0404 (3)	
N2B	0.20709 (6)	0.43498 (10)	0.34187 (6)	0.0389 (3)	
N3B	0.21983 (6)	0.42506 (11)	0.54935 (6)	0.0423 (3)	
C1B	0.31671 (7)	0.41479 (11)	0.49376 (6)	0.0327 (3)	
C2B	0.33981 (7)	0.41348 (11)	0.43547 (6)	0.0328 (3)	
C3B	0.30566 (7)	0.41716 (10)	0.38727 (6)	0.0323 (3)	
H3B	0.3236	0.4144	0.3514	0.039*	
C4B	0.24333 (7)	0.42513 (11)	0.39268 (6)	0.0323 (3)	
C5B	0.21633 (7)	0.42567 (11)	0.44617 (7)	0.0331 (3)	
H5B	0.1745	0.4287	0.4493	0.040*	
C6B	0.25131 (7)	0.42169 (11)	0.49451 (6)	0.0328 (3)	

### Atomic displacement parameters (Å^2^)

**Table:**

	*U*^11^	*U*^22^	*U*^33^	*U*^12^	*U*^13^	*U*^23^
S1	0.0416 (2)	0.0577 (2)	0.03319 (19)	−0.01666 (18)	0.00612 (15)	−0.00591 (17)
N1	0.0354 (7)	0.0518 (8)	0.0288 (6)	−0.0032 (6)	−0.0028 (5)	−0.0044 (6)
N2	0.0365 (6)	0.0401 (7)	0.0279 (6)	−0.0006 (5)	0.0053 (5)	−0.0001 (5)
N3	0.0261 (6)	0.0449 (7)	0.0284 (6)	−0.0014 (5)	0.0050 (5)	−0.0049 (5)
N4	0.0336 (6)	0.0504 (7)	0.0261 (6)	−0.0097 (6)	0.0026 (5)	0.0029 (5)
C1	0.0472 (9)	0.0447 (8)	0.0296 (7)	−0.0103 (7)	0.0022 (6)	−0.0017 (6)
C2	0.0404 (8)	0.0406 (8)	0.0269 (7)	−0.0048 (6)	−0.0004 (6)	−0.0003 (6)
C3	0.0322 (7)	0.0381 (7)	0.0278 (7)	0.0014 (6)	0.0003 (6)	−0.0004 (6)
C4	0.0352 (7)	0.0415 (8)	0.0275 (7)	−0.0009 (6)	0.0013 (6)	0.0007 (6)
C5	0.0479 (9)	0.0413 (8)	0.0232 (7)	0.0005 (7)	−0.0022 (6)	0.0017 (6)
C6	0.0662 (11)	0.0441 (9)	0.0312 (8)	−0.0058 (8)	−0.0053 (8)	0.0003 (7)
C7	0.0919 (15)	0.0391 (9)	0.0446 (10)	0.0011 (9)	−0.0001 (10)	−0.0036 (8)
C8	0.0820 (14)	0.0447 (10)	0.0541 (11)	0.0180 (10)	0.0118 (10)	−0.0040 (8)
C9	0.0531 (10)	0.0491 (10)	0.0438 (9)	0.0109 (8)	0.0071 (8)	−0.0005 (8)
C10	0.0465 (8)	0.0386 (8)	0.0264 (7)	0.0045 (7)	0.0019 (6)	0.0002 (6)
C11	0.0271 (6)	0.0403 (8)	0.0256 (6)	0.0047 (6)	−0.0017 (5)	0.0009 (6)
C12	0.0237 (6)	0.0583 (9)	0.0284 (7)	−0.0046 (6)	0.0028 (5)	−0.0101 (7)
C13	0.0333 (7)	0.0660 (10)	0.0238 (7)	−0.0108 (7)	0.0051 (6)	−0.0042 (7)
C14	0.0276 (7)	0.0458 (8)	0.0304 (7)	−0.0043 (6)	0.0031 (6)	−0.0002 (6)
C15	0.0272 (6)	0.0424 (8)	0.0264 (7)	−0.0025 (6)	0.0054 (5)	−0.0033 (6)
C16	0.0580 (11)	0.0583 (11)	0.0458 (10)	−0.0085 (9)	0.0077 (8)	0.0159 (8)
C17	0.0638 (11)	0.0657 (11)	0.0314 (8)	−0.0231 (9)	0.0097 (8)	−0.0027 (8)
O1A	0.0613 (7)	0.0564 (7)	0.0306 (6)	−0.0125 (6)	0.0103 (5)	−0.0060 (5)
O2AA	0.0631 (12)	0.106 (2)	0.0235 (7)	0.0052 (13)	−0.0053 (7)	0.0046 (10)
O2AB	0.0631 (12)	0.106 (2)	0.0235 (7)	0.0052 (13)	−0.0053 (7)	0.0046 (10)
O3A	0.0481 (7)	0.0840 (10)	0.0447 (7)	−0.0176 (7)	−0.0127 (6)	0.0023 (6)
O4A	0.0556 (7)	0.0495 (7)	0.0391 (6)	−0.0013 (5)	0.0091 (5)	0.0018 (5)
O5A	0.0711 (9)	0.0933 (11)	0.0253 (6)	−0.0010 (8)	−0.0083 (6)	0.0001 (6)
O6A	0.0511 (8)	0.1067 (12)	0.0609 (9)	0.0217 (8)	−0.0123 (7)	0.0111 (8)
O7A	0.0458 (7)	0.1042 (12)	0.0608 (9)	−0.0024 (8)	0.0038 (6)	0.0181 (8)
N1A	0.0582 (9)	0.0472 (8)	0.0299 (7)	−0.0005 (7)	−0.0116 (6)	−0.0024 (6)
N2A	0.0566 (8)	0.0317 (6)	0.0271 (6)	0.0002 (6)	−0.0013 (6)	0.0008 (5)
N3A	0.0446 (8)	0.0480 (8)	0.0464 (8)	0.0113 (6)	−0.0022 (6)	−0.0015 (6)
C1A	0.0499 (9)	0.0279 (7)	0.0270 (7)	−0.0042 (6)	0.0019 (6)	0.0008 (5)
C2A	0.0473 (8)	0.0275 (7)	0.0259 (7)	−0.0025 (6)	−0.0068 (6)	0.0017 (5)
C3A	0.0431 (8)	0.0228 (6)	0.0308 (7)	−0.0015 (6)	−0.0028 (6)	0.0010 (5)
C4A	0.0494 (8)	0.0231 (6)	0.0251 (7)	0.0008 (6)	−0.0019 (6)	0.0009 (5)
C5A	0.0483 (8)	0.0288 (7)	0.0294 (7)	0.0067 (6)	−0.0087 (6)	0.0000 (6)
C6A	0.0422 (8)	0.0304 (7)	0.0349 (8)	0.0043 (6)	−0.0022 (6)	0.0010 (6)
O1B	0.0393 (6)	0.0569 (7)	0.0357 (6)	0.0006 (5)	−0.0067 (5)	−0.0067 (5)
O2B	0.0372 (6)	0.0820 (9)	0.0716 (9)	−0.0158 (6)	0.0051 (6)	−0.0263 (8)
O3B	0.0432 (6)	0.0829 (10)	0.0509 (7)	0.0141 (6)	0.0049 (6)	−0.0162 (7)
O4B	0.0610 (7)	0.0525 (7)	0.0350 (6)	0.0002 (6)	−0.0071 (5)	−0.0003 (5)
O5B	0.0428 (6)	0.0530 (7)	0.0569 (7)	0.0108 (5)	−0.0142 (5)	−0.0093 (6)
O6B	0.0421 (7)	0.0905 (10)	0.0601 (8)	−0.0069 (7)	0.0161 (6)	0.0031 (7)
O7B	0.0554 (7)	0.0927 (10)	0.0340 (6)	0.0016 (7)	0.0070 (6)	−0.0038 (7)
N1B	0.0343 (6)	0.0458 (7)	0.0409 (7)	0.0008 (6)	0.0028 (6)	−0.0014 (6)
N2B	0.0466 (7)	0.0274 (6)	0.0426 (7)	0.0033 (5)	−0.0101 (6)	−0.0039 (5)
N3B	0.0397 (7)	0.0468 (8)	0.0405 (7)	0.0033 (6)	0.0083 (6)	0.0042 (6)
C1B	0.0346 (7)	0.0293 (7)	0.0343 (7)	−0.0022 (6)	−0.0002 (6)	−0.0027 (6)
C2B	0.0308 (7)	0.0295 (7)	0.0382 (8)	−0.0015 (6)	0.0020 (6)	−0.0019 (6)
C3B	0.0400 (8)	0.0246 (6)	0.0323 (7)	−0.0007 (6)	0.0026 (6)	−0.0013 (5)
C4B	0.0389 (7)	0.0225 (6)	0.0356 (7)	0.0001 (5)	−0.0051 (6)	−0.0007 (5)
C5B	0.0309 (7)	0.0246 (7)	0.0439 (8)	0.0007 (5)	−0.0004 (6)	−0.0008 (6)
C6B	0.0348 (7)	0.0292 (7)	0.0343 (7)	−0.0003 (6)	0.0037 (6)	0.0004 (6)

### Geometric parameters (Å, º)

**Table:**

S1—C4	1.7157 (15)	C16—H16B	0.9600
S1—C1	1.7362 (16)	C16—H16C	0.9600
N1—C4	1.3935 (19)	C17—H17A	0.9600
N1—C5	1.420 (2)	C17—H17B	0.9600
N1—H1	0.852 (19)	C17—H17C	0.9600
N2—C11	1.3295 (19)	O1A—C1A	1.2432 (18)
N2—C10	1.426 (2)	O2AA—N1A	1.261 (2)
N2—H2	0.872 (17)	O2AB—N1A	1.158 (7)
N3—C11	1.336 (2)	O3A—N1A	1.230 (2)
N3—C15	1.4690 (18)	O4A—N2A	1.2184 (18)
N3—C12	1.4788 (18)	O5A—N2A	1.2244 (18)
N4—C16	1.492 (2)	O6A—N3A	1.2212 (19)
N4—C13	1.494 (2)	O7A—N3A	1.209 (2)
N4—C14	1.4958 (18)	N1A—C2A	1.4528 (19)
N4—H4	0.926 (19)	N2A—C4A	1.4401 (19)
C1—C2	1.348 (2)	N3A—C6A	1.461 (2)
C1—C17	1.499 (2)	C1A—C2A	1.449 (2)
C2—C3	1.440 (2)	C1A—C6A	1.453 (2)
C2—H2A	0.9300	C2A—C3A	1.375 (2)
C3—C4	1.377 (2)	C3A—C4A	1.384 (2)
C3—C11	1.453 (2)	C3A—H3A	0.9300
C5—C10	1.388 (2)	C4A—C5A	1.390 (2)
C5—C6	1.391 (2)	C5A—C6A	1.359 (2)
C6—C7	1.386 (3)	C5A—H5A	0.9300
C6—H6	0.9300	O1B—C1B	1.2383 (18)
C7—C8	1.371 (3)	O2B—N1B	1.2201 (19)
C7—H7	0.9300	O3B—N1B	1.2219 (18)
C8—C9	1.387 (3)	O4B—N2B	1.2291 (18)
C8—H8	0.9300	O5B—N2B	1.2363 (18)
C9—C10	1.384 (2)	O6B—N3B	1.2233 (19)
C9—H9	0.9300	O7B—N3B	1.2264 (18)
C12—C13	1.510 (2)	N1B—C2B	1.4599 (19)
C12—H12A	0.9700	N2B—C4B	1.4401 (19)
C12—H12B	0.9700	N3B—C6B	1.4613 (19)
C13—H13A	0.9700	C1B—C6B	1.452 (2)
C13—H13B	0.9700	C1B—C2B	1.457 (2)
C14—C15	1.506 (2)	C2B—C3B	1.359 (2)
C14—H14A	0.9700	C3B—C4B	1.391 (2)
C14—H14B	0.9700	C3B—H3B	0.9300
C15—H15A	0.9700	C4B—C5B	1.387 (2)
C15—H15B	0.9700	C5B—C6B	1.372 (2)
C16—H16A	0.9600	C5B—H5B	0.9300
			
C4—S1—C1	92.47 (7)	C14—C15—H15B	109.5
C4—N1—C5	113.83 (13)	H15A—C15—H15B	108.1
C4—N1—H1	113.2 (13)	N4—C16—H16A	109.5
C5—N1—H1	113.3 (14)	N4—C16—H16B	109.5
C11—N2—C10	127.92 (13)	H16A—C16—H16B	109.5
C11—N2—H2	119.2 (11)	N4—C16—H16C	109.5
C10—N2—H2	112.8 (11)	H16A—C16—H16C	109.5
C11—N3—C15	123.57 (12)	H16B—C16—H16C	109.5
C11—N3—C12	124.95 (12)	C1—C17—H17A	109.5
C15—N3—C12	111.39 (12)	C1—C17—H17B	109.5
C16—N4—C13	112.37 (13)	H17A—C17—H17B	109.5
C16—N4—C14	110.72 (13)	C1—C17—H17C	109.5
C13—N4—C14	110.11 (12)	H17A—C17—H17C	109.5
C16—N4—H4	108.6 (12)	H17B—C17—H17C	109.5
C13—N4—H4	110.4 (12)	O2AB—N1A—O3A	112.2 (4)
C14—N4—H4	104.4 (12)	O3A—N1A—O2AA	124.15 (16)
C2—C1—C17	130.90 (15)	O2AB—N1A—C2A	124.2 (4)
C2—C1—S1	110.58 (11)	O3A—N1A—C2A	118.11 (13)
C17—C1—S1	118.52 (12)	O2AA—N1A—C2A	116.98 (16)
C1—C2—C3	113.99 (14)	O4A—N2A—O5A	123.05 (14)
C1—C2—H2A	123.0	O4A—N2A—C4A	119.28 (13)
C3—C2—H2A	123.0	O5A—N2A—C4A	117.66 (14)
C4—C3—C2	111.45 (13)	O7A—N3A—O6A	122.79 (16)
C4—C3—C11	121.03 (13)	O7A—N3A—C6A	118.90 (14)
C2—C3—C11	127.40 (13)	O6A—N3A—C6A	118.27 (15)
C3—C4—N1	126.67 (14)	O1A—C1A—C2A	125.21 (14)
C3—C4—S1	111.47 (11)	O1A—C1A—C6A	122.84 (15)
N1—C4—S1	121.76 (12)	C2A—C1A—C6A	111.82 (13)
C10—C5—C6	119.69 (16)	C3A—C2A—C1A	123.79 (13)
C10—C5—N1	118.46 (14)	C3A—C2A—N1A	116.09 (14)
C6—C5—N1	121.85 (16)	C1A—C2A—N1A	120.09 (13)
C7—C6—C5	119.68 (18)	C2A—C3A—C4A	118.97 (14)
C7—C6—H6	120.2	C2A—C3A—H3A	120.5
C5—C6—H6	120.2	C4A—C3A—H3A	120.5
C8—C7—C6	120.31 (18)	C3A—C4A—C5A	121.11 (14)
C8—C7—H7	119.8	C3A—C4A—N2A	119.07 (14)
C6—C7—H7	119.8	C5A—C4A—N2A	119.81 (13)
C7—C8—C9	120.45 (18)	C6A—C5A—C4A	119.35 (14)
C7—C8—H8	119.8	C6A—C5A—H5A	120.3
C9—C8—H8	119.8	C4A—C5A—H5A	120.3
C10—C9—C8	119.60 (18)	C5A—C6A—C1A	123.83 (15)
C10—C9—H9	120.2	C5A—C6A—N3A	117.11 (14)
C8—C9—H9	120.2	C1A—C6A—N3A	119.05 (14)
C9—C10—C5	120.22 (16)	O2B—N1B—O3B	123.44 (14)
C9—C10—N2	117.83 (15)	O2B—N1B—C2B	118.47 (13)
C5—C10—N2	121.51 (14)	O3B—N1B—C2B	118.04 (13)
N2—C11—N3	119.39 (13)	O4B—N2B—O5B	123.21 (14)
N2—C11—C3	119.48 (14)	O4B—N2B—C4B	118.47 (13)
N3—C11—C3	121.12 (13)	O5B—N2B—C4B	118.32 (13)
N3—C12—C13	111.76 (12)	O6B—N3B—O7B	123.05 (15)
N3—C12—H12A	109.3	O6B—N3B—C6B	117.82 (14)
C13—C12—H12A	109.3	O7B—N3B—C6B	119.13 (13)
N3—C12—H12B	109.3	O1B—C1B—C6B	126.23 (14)
C13—C12—H12B	109.3	O1B—C1B—C2B	122.46 (13)
H12A—C12—H12B	107.9	C6B—C1B—C2B	111.27 (13)
N4—C13—C12	111.10 (12)	C3B—C2B—C1B	125.50 (13)
N4—C13—H13A	109.4	C3B—C2B—N1B	116.48 (13)
C12—C13—H13A	109.4	C1B—C2B—N1B	117.99 (13)
N4—C13—H13B	109.4	C2B—C3B—C4B	118.70 (14)
C12—C13—H13B	109.4	C2B—C3B—H3B	120.6
H13A—C13—H13B	108.0	C4B—C3B—H3B	120.6
N4—C14—C15	110.66 (12)	C5B—C4B—C3B	120.72 (13)
N4—C14—H14A	109.5	C5B—C4B—N2B	120.24 (14)
C15—C14—H14A	109.5	C3B—C4B—N2B	119.03 (13)
N4—C14—H14B	109.5	C6B—C5B—C4B	119.97 (14)
C15—C14—H14B	109.5	C6B—C5B—H5B	120.0
H14A—C14—H14B	108.1	C4B—C5B—H5B	120.0
N3—C15—C14	110.55 (12)	C5B—C6B—C1B	123.80 (14)
N3—C15—H15A	109.5	C5B—C6B—N3B	116.92 (13)
C14—C15—H15A	109.5	C1B—C6B—N3B	119.28 (13)
N3—C15—H15B	109.5		
			
C4—S1—C1—C2	0.61 (14)	O3A—N1A—C2A—C3A	−2.0 (2)
C4—S1—C1—C17	−179.44 (15)	O2AA—N1A—C2A—C3A	168.4 (2)
C17—C1—C2—C3	178.40 (18)	O2AB—N1A—C2A—C1A	24.2 (6)
S1—C1—C2—C3	−1.65 (19)	O3A—N1A—C2A—C1A	176.02 (14)
C1—C2—C3—C4	2.1 (2)	O2AA—N1A—C2A—C1A	−13.6 (3)
C1—C2—C3—C11	−173.89 (15)	C1A—C2A—C3A—C4A	4.0 (2)
C2—C3—C4—N1	174.83 (15)	N1A—C2A—C3A—C4A	−178.08 (13)
C11—C3—C4—N1	−8.8 (2)	C2A—C3A—C4A—C5A	3.5 (2)
C2—C3—C4—S1	−1.61 (17)	C2A—C3A—C4A—N2A	−177.38 (12)
C11—C3—C4—S1	174.72 (11)	O4A—N2A—C4A—C3A	1.3 (2)
C5—N1—C4—C3	−58.5 (2)	O5A—N2A—C4A—C3A	−179.14 (14)
C5—N1—C4—S1	117.65 (14)	O4A—N2A—C4A—C5A	−179.57 (13)
C1—S1—C4—C3	0.61 (13)	O5A—N2A—C4A—C5A	0.0 (2)
C1—S1—C4—N1	−176.03 (14)	C3A—C4A—C5A—C6A	−2.7 (2)
C4—N1—C5—C10	58.27 (18)	N2A—C4A—C5A—C6A	178.21 (13)
C4—N1—C5—C6	−121.34 (16)	C4A—C5A—C6A—C1A	−5.7 (2)
C10—C5—C6—C7	2.3 (2)	C4A—C5A—C6A—N3A	175.64 (13)
N1—C5—C6—C7	−178.14 (15)	O1A—C1A—C6A—C5A	−164.27 (15)
C5—C6—C7—C8	−2.7 (3)	C2A—C1A—C6A—C5A	11.8 (2)
C6—C7—C8—C9	1.3 (3)	O1A—C1A—C6A—N3A	14.4 (2)
C7—C8—C9—C10	0.5 (3)	C2A—C1A—C6A—N3A	−169.53 (13)
C8—C9—C10—C5	−0.9 (2)	O7A—N3A—C6A—C5A	−152.39 (16)
C8—C9—C10—N2	171.61 (16)	O6A—N3A—C6A—C5A	25.2 (2)
C6—C5—C10—C9	−0.5 (2)	O7A—N3A—C6A—C1A	28.9 (2)
N1—C5—C10—C9	179.92 (14)	O6A—N3A—C6A—C1A	−153.59 (16)
C6—C5—C10—N2	−172.70 (14)	O1B—C1B—C2B—C3B	176.98 (14)
N1—C5—C10—N2	7.7 (2)	C6B—C1B—C2B—C3B	−0.6 (2)
C11—N2—C10—C9	133.74 (16)	O1B—C1B—C2B—N1B	−4.9 (2)
C11—N2—C10—C5	−53.9 (2)	C6B—C1B—C2B—N1B	177.57 (12)
C10—N2—C11—N3	−168.35 (14)	O2B—N1B—C2B—C3B	137.08 (16)
C10—N2—C11—C3	12.7 (2)	O3B—N1B—C2B—C3B	−40.4 (2)
C15—N3—C11—N2	−159.95 (13)	O2B—N1B—C2B—C1B	−41.2 (2)
C12—N3—C11—N2	16.3 (2)	O3B—N1B—C2B—C1B	141.27 (15)
C15—N3—C11—C3	19.0 (2)	C1B—C2B—C3B—C4B	1.5 (2)
C12—N3—C11—C3	−164.74 (13)	N1B—C2B—C3B—C4B	−176.69 (12)
C4—C3—C11—N2	36.9 (2)	C2B—C3B—C4B—C5B	−2.2 (2)
C2—C3—C11—N2	−147.44 (15)	C2B—C3B—C4B—N2B	176.57 (12)
C4—C3—C11—N3	−142.10 (15)	O4B—N2B—C4B—C5B	177.81 (13)
C2—C3—C11—N3	33.6 (2)	O5B—N2B—C4B—C5B	−1.8 (2)
C11—N3—C12—C13	−121.45 (15)	O4B—N2B—C4B—C3B	−0.97 (19)
C15—N3—C12—C13	55.18 (15)	O5B—N2B—C4B—C3B	179.42 (13)
C16—N4—C13—C12	178.99 (13)	C3B—C4B—C5B—C6B	2.1 (2)
C14—N4—C13—C12	55.07 (16)	N2B—C4B—C5B—C6B	−176.65 (13)
N3—C12—C13—N4	−54.21 (16)	C4B—C5B—C6B—C1B	−1.2 (2)
C16—N4—C14—C15	177.80 (13)	C4B—C5B—C6B—N3B	178.58 (13)
C13—N4—C14—C15	−57.33 (15)	O1B—C1B—C6B—C5B	−177.01 (14)
C11—N3—C15—C14	119.67 (14)	C2B—C1B—C6B—C5B	0.4 (2)
C12—N3—C15—C14	−57.02 (15)	O1B—C1B—C6B—N3B	3.2 (2)
N4—C14—C15—N3	58.41 (15)	C2B—C1B—C6B—N3B	−179.37 (12)
O1A—C1A—C2A—C3A	165.04 (15)	O6B—N3B—C6B—C5B	19.5 (2)
C6A—C1A—C2A—C3A	−10.9 (2)	O7B—N3B—C6B—C5B	−159.31 (15)
O1A—C1A—C2A—N1A	−12.8 (2)	O6B—N3B—C6B—C1B	−160.69 (15)
C6A—C1A—C2A—N1A	171.21 (13)	O7B—N3B—C6B—C1B	20.5 (2)
O2AB—N1A—C2A—C3A	−153.8 (6)		

### Hydrogen-bond geometry (Å, º)

**Table:**

*D*—H···*A*	*D*—H	H···*A*	*D*···*A*	*D*—H···*A*
N2—H2···O1*A*	0.872 (17)	1.848 (17)	2.7086 (17)	168.5 (16)
N4—H4···O1*B*	0.926 (19)	1.859 (19)	2.6612 (17)	143.6 (17)
N4—H4···O7*B*	0.926 (19)	2.449 (19)	3.1714 (19)	135.0 (15)
N1—H1···O5*B*^i^	0.852 (19)	2.40 (2)	3.1395 (18)	145.9 (17)
C5*B*—H5*B*···S1^ii^	0.93	2.87	3.6225 (15)	139
C8—H8···O3*A*^iii^	0.93	2.48	3.349 (2)	156
C12—H12*A*···O5*A*^iv^	0.97	2.23	3.0970 (19)	149
C17—H17*B*···O6*A*^iv^	0.96	2.43	3.207 (2)	138

Symmetry codes: (i) *x*+1/2, −*y*+3/2, −*z*+1; (ii) *x*−1/2, −*y*+3/2, −*z*+1; (iii) −*x*+1/2, *y*+1/2, *z*; (iv) *x*, −*y*+3/2, *z*−1/2.
